# Exogenous betaine enhances salt tolerance of *Glycyrrhiza uralensis* through multiple pathways

**DOI:** 10.1186/s12870-024-04851-w

**Published:** 2024-03-02

**Authors:** Xinping Dong, Xiaomei Ma, Zhilong Zhao, Miao Ma

**Affiliations:** 1 College of Life Sciences, Shihezi University, Shihezi, 832003 China; 2 Ministry of Education Key Laboratory of Xinjiang Phytomedicine Resource Utilization, College of Life Sciences, Shihezi University, Shihezi, 832003 China

**Keywords:** Liquorice, Salinity, Salt secretion, Physiological mechanism, Glycine betaine

## Abstract

**Background:**

*Glycyrrhiza uralensis* Fisch., a valuable medicinal plant, shows contrasting salt tolerance between seedlings and perennial individuals, and salt tolerance at seedling stage is very weak. Understanding this difference is crucial for optimizing cultivation practices and maximizing the plant’s economic potential. Salt stress resistance at the seedling stage is the key to the cultivation of the plant using salinized land. This study investigated the physiological mechanism of the application of glycine betaine (0, 10, 20, 40, 80 mM) to seedling stages of *G. uralensis* under salt stress (160 mM NaCl).

**Results:**

*G. uralensis* seedlings’ growth was severely inhibited under NaCl stress conditions, but the addition of GB effectively mitigated its effects, with 20 mM GB had showing most significant alleviating effect. The application of 20 mM GB under NaCl stress conditions significantly increased total root length (80.38%), total root surface area (93.28%), and total root volume (175.61%), and significantly increased the GB content in its roots, stems, and leaves by 36.88%, 107.05%, and 21.63%, respectively. The activity of betaine aldehyde dehydrogenase 2 (BADH_2_) was increased by 74.10%, 249.38%, and 150.60%, respectively. The 20 mM GB-addition treatment significantly increased content of osmoregulatory substances (the contents of soluble protein, soluble sugar and proline increased by 7.05%, 70.52% and 661.06% in roots, and also increased by 30.74%, 47.11% and 26.88% in leaves, respectively.). Furthermore, it markedly enhanced the activity of antioxidant enzymes and the content of antioxidants (SOD, CAT, POD, APX and activities and ASA contents were elevated by 59.55%, 413.07%, 225.91%, 300.00% and 73.33% in the root, and increased by 877.51%, 359.89%, 199.15%, 144.35%, and 108.11% in leaves, respectively.), and obviously promoted salt secretion capacity of the leaves, which especially promoted the secretion of Na^+^ (1.37 times).

**Conclusions:**

In summary, the exogenous addition of GB significantly enhances the salt tolerance of *G. uralensis* seedlings, promoting osmoregulatory substances, antioxidant enzyme activities, excess salt discharge especially the significant promotion of the secretion of Na^+^Future studies should aim to elucidate the molecular mechanisms that operate when GB regulates saline stress tolerance.

## Background

Soil salinization is a major factor limiting crop yields globally (especially in drylands) [1]. According to the Food and Agriculture Organization of the United Nations, the global salinized land area was approximately 8.33 × 10^8^ hm^2^ in 2021, and there was a salinized land area of approximately 9.9 × 10^7^ hm^2^, in China [2]. Salt stress (mainly NaCl stress) triggers osmotic imbalance, excessive accumulation of reactive oxygen species (ROS) and Na^+^ retention through osmotic stress, oxidative stress, and ionic toxicity [3–5]. They disrupt plant metabolic systems, lead to reduction in crop yield and quality crop, and substantial losses in the agricultural economy. Due to limited arable land, exploiting salt-tolerant plants and improving their salt tolerance is important to develop the economy of saline soils and to increase farmers’ income, which is also a hot topic in research presently.

*Glycyrrhiza uralensis* Fisch. is a halophyte of Leguminosae and has a high commercial value. Its dried roots and rhizomes are traditionally used as herbal medicines due to its flavonoids and triterpenoids components [6]. Its stems and leaves are high in crude protein and crude fat and low in crude fiber; thus, the liquorice is an excellent forage or supplementary grass for cattle and sheep [7]. Notably, as increasing of people’s health awareness, natural medicine use has risen rapidly worldwide, and the demand for *G. uralensis* in the world has increased; thus, the dwindling wild liquorice resources cannot fulfil market demand [8, 9]. Therefore, artificial cultivation of the plant should be implemented to solve the imbalance between supply and demand in the liquorice industry [9]. Most liquorice planting areas are threatened by soil salinization, especially in the northern part of China. Although adult individuals of *G. uralensis* have strong salt tolerance, the tolerance is relatively weak in their seedling stage [10]. Planting of *G. uralensis* in salinized land, dead seedlings often occurred [11], the medicinal material’s production was reduced sustainable sharply, which restricted the development of the liquorice industry. Studies have shown that NaCl concentrations exceeding 150 mM inhibited the growth, development, and quality of licorice, ultimately leading to a decrease in its medicinal and economic value [12–14]. Notably, the history of liquorice from a wild to a cultivated plant is relatively short compared to other crops [15, 16], and the breeding for germplasm with strong salt tolerance has not occurred. Therefore, improving the salt tolerance of *G. uralensis* by cultivation techniques is urgently necessary. Addition of exogenous substances has become one of the most effective cultivation techniques to improve salt tolerance of crops in a short time [17, 18]. Glycine belaine (GB) is a kind of growth regulator widely present in organisms. Exogenous GB is chemically and physically stable, and is easily absorbed by plants, and its synthesis process has been well developed [19, 20]. The literature demonstrated that exogenous GB can enhance the activity of enzymatic and non-enzymatic systems, reduce ROS (reactive oxygen species) accumulation, and increase the content of osmoregulatory substances to enhance the salt tolerance of plants [21, 22]. Exogenous GB can significantly enhance salt tolerance and significantly increase fruit or seed yield of cotton (*Gossypium hirsutum* L.) [18], maize (*Zea mays*) [23], rice (*Oryza sativa*) [24], and tomato (*Lycopersicon esculentum*) [25]. However, further research is necessary on whether the GB may improve the root yield of a plant.

Even though various studies have been conducted on the effects of salinity stress on *G. uralensis* seedlings, however, the effect of GB application has largely been overlooked. We hypothesized that exogenous GB might plant growth and metabolism by mitigating the adverse impacts of Salinity. We used controlled and NaCl stress conditions (160 mM NaCl) with exogenous GB application (0, 10, 20, 40 and 80 mM GB) to test our hypothesis via a morpho-physio-biochemical investigation. Our aim was to gain deeper understanding of the effects of GB addition on salt tolerance indices of *G. uralensis* seedlings under NaCl stress conditions, such as (1) growth and biomass, (2) osmotic adjustment, (3) reactive oxygen metabolism system, and (4) salt secretion. This study was to provide a theoretical basis and technical support for the cultivation of high-yield and high-quality liquorice herbs in salinized land.

## Material and methods

### Plant material and experimental design

The *G. uralensis* seeds were provided by the Institute of Liquorice in Shihezi University. The experiment was conducted from April to October in 2021 on the campus of Shihezi University, China (44° 31′ 47″ N, 86° 06′ 28″ E). Healthy and full seeds with the same size were selected and soaked in 98% sulfuric acid solution at room temperature (25℃) for 30 min, washed with tap water until there was no sulfuric acid residue on their surface, and then soaked in distilled water for 12 h. Next, the seeds were spread evenly in the plastic pot (bottom diameter × top diameter × height = 20 cm × 30 cm × 20 cm; gauze mesh was laid at the bottom). Each pot contained approximately 8 kg sandy soil, and the ratio of river sand to loam was 7:3. The soil background details are presented in Table 1, 15 seeds were evenly sown in each pot at a sowing depth of 1 cm.

**Table 1:** Physical and chemical properties of sandy soil

Total nitrogen g/kg	Total phosphorus g/kg	Total potassium g/kg	Available N mg/kg	Available P mg/kg	Available K mg/kg	Organic matter g/kg
0.315	0.131	5.47	52.59	5.23	50.04	6.64

Fertilizing sscheme were similar to those of *G. uralensis* field management (urea [N ≥ 46%] 14.99 g/m^2^, calcium superphosphate [P_2_O_5_ ≥ 46%] 23.99 g/m^2^, potassium sulfate [K_2_O ≥ 50%] 10.49 g/m^2^) [26], of which calcium superphosphate was applied as a basal fertilizer, one-third of the urea and potassium sulfate were also applied as basal fertilizers. The remaining two-thirds of them were applied at the seedling stage. When the *G. uralensis* seedlings had six true leaves, four seedlings of uniform growth were retained in each pot by converting the planting density of cultivated liquorice fields (usually 6.0 × 10^5^ plants/hm^2^) with the area of the upper calibration of the plastic pots.

NaCl concentration was set by referring to the salinity range of *G. uralensis* [27], 200 mL NaCl solution was used for watering every other day for a total of 15 times. Once the salt treatment was over, 200 mL GB (C_5_H_11_NC_2_ was purchased from McLean Company and had a relative molecular mass of 118.15 and a purity greater than 98%) solution of the corresponding concentration (10, 20, 40, and 80 mM GB) was applied to the roots every 5 d, for a total of three times; an equal amount of distilled water was applied to the control group and NaCl-only treatment group [28].

The detailed formulation of each treatment group is shown in Table 2. Each treatment was replicated five times. Except for the experimental treatments, the other management measures used were the same as those of the *G. uralensis* field. Samples were collected at 9:00 a.m. every day on the seventh day after the last treatment. Next, the corresponding indices were measured. The third fully expanded functional leaf (from the top of the plant) was used to determine the physiological indices. Plants harvested and measured their biomass after all physiological indices had been measured.

**Table 2:** Experimental treatments

Code	Treatments abbreviations	NaCl concentration (mM)	GB concentration (mM)
(1)	CK	0	0
(2)	NaCl	160	0
(3)	NaCl + GB10	160	10
(4)	NaCl + GB20	160	20
(5)	NaCl + GB40	160	40
(6)	NaCl + GB80	160	80

### Plant growth parameters

#### Height

The height of the plant was measured from the surface of the potting soil to the terminal bud of the plant. Ten plants were randomly selected for each treatment.

#### Root morphological indices

The root systems of above plants were placed in an acrylic tray (25 cm × 15 cm) of a root scanner (WinRHIZO La 2400, Epson, Japan), and was scanned using EPSON Scan scanning software. After obtaining the scanned images of the root system, the root system analysis system WinRHIZO was used to measure the root morphology indices such as total root length (TRL), total root surface area (TRSA), and total root volume (TRV) and other morphological indicators of the root system.

#### Biomass

Ten individual plants were randomly selected from each treatment. The roots were removed from the plastic pot, and the sandy soil on the surface of the roots was gently washed with tap water. The roots were divided into three parts (roots, stems, and leaves), they were placed into a paper bag, heated in an oven at 105 ℃ for 30 min, and then dried at 80 ℃ to a constant weight. The dry weight was determined by an analytical balance with an accuracy of 0.001 g (BS423S, Sartorius, Germany).

### Determination of endogenous betaine content and betaine aldehyde dehydrogenase activity

The content of GB (endogenous betaine) and the activity of BADH_2_ (betaine aldehyde dehydrogenase) in roots, leaves, and stems of *G. uralensis* were determined by using a Solarbio kit (GB-BC3130, Beijing Solarbio Science and Technology Co., Ltd., China) and a botanical betaine aldehyde dehydrogenase 2 (BADH_2_) ELISA kit (JM-110368P2, Jiangsu Jingmei Biological Technology Co., Ltd., China), respectively. A spectrophotometer was used to measure GB and BADH_2_ absorbance at 525 nm and 450 nm, respectively.

### Determination of osmoregulatory substances

Proline (Pro), soluble sugar (SS), and soluble protein (SP) contents in the roots and leaves of *G. uralensis* were determined using the acidic indinotrione method [29], anthrone method [30], and Coomassie brilliant blue (G-250) method [31], respectively. The absorbance of proline, soluble sugar and soluble protein at 520, 620 and 595 nm was read by spectrophotometer.

### Determination of conductivity and malondialdehyde content

The relative conductivity of the roots and leaves of *G. uralensis* was determined using the conductivity method [32]. One gram of fresh leaves was placed in a 15 mL centrifuge tube, to which 10 mL deionised water was added. The leaves were then soaked at room temperature for 12 h. The conductivity of the solution was measured using a conductivity meter (Bante 5, Shanghai Bante, China) and recorded as R_1_. Next, it was heated in a water bath at 100 °C for 30 min. Its conductivity was measured after the solution was cooled to room temperature and recorded as R_2_. The formula for calculating the relative electrical conductivity is as follows:

$$\mathrm{EL}={\mathrm R}_1/{\mathrm R}_2\times100\%$$

For the evaluation of lipid peroxidation, malondialdehyde (MDA) concentrations were determined using thiobarbital acid method [33]. The absorbance at 450, 532, and 600 nm were read using a spectrophotometer; the concentrations of MDA werecalculated using the following equation:

$$\mathrm{MDA}(\mathrm{mol}\;\mathrm g^{-1}\;\mathrm{FW})=6.45(A532-A600)-0.56A450$$

### Determination of reactive oxygen scavenging systems

Hydrogen peroxide (H_2_O_2_) and superoxide anions (O_2_^−^^.^) content and superoxide dismutase (SOD), peroxidase (POD), catalase (CAT) and ascorbate peroxidase (APX) activity in the leaves and roots of *G. uralensis* were measured using Solarbio kits (H_2_O_2_-BC3590, O_2_^−^^.^-BC1290, SOD-BC0170, POD-BC0095, CAT-C0200, APX-BC0220, Beijing Solarbio Technology Co., Ltd., China). Above indicators were calculated by reading the absorbance at 415 nm, 530 nm, 560 nm, 470 nm, 240 nm and 290 nm. Ascorbic acid content (ASA) was quantitatively determined by using the 2,6-dichlorophenol indidophenol (DCPIP) method [34].

### Observation of salt discharge behaviour and ionic measurements

The stomata and salt glands on the lower leaves of *G. uralensis* have strong salt secretion ability [35]. Therefore, some lower healthy and fully expanded leaves were selected, cut into 0.5 cm × 0.5 cm pieces (over the main vein) near the apical one-third of the leaf, and fixed in FAA for 48 h. Next, the fixed material was glued to the sample stage and subjected to ion sputtering plating. An Electron microscope (SU8010, Hitachi High-tech Company, Japan) was used to observe secreted salt by the salt glands and stomatal of *G. uralensis* and capture images. Each treatment was repeated for five materials.

Referring to Newete [36], the experimental material was placed under a rain-proof shed, and 15 fully expanded lower leaves were randomly selected. The upper and lower surfaces of the leaves were cleaned with deionised water and then marked. After 7 d, the leaves were placed in a centrifuge tube containing 20 mL ultrapure water, centrifuged at 2000 r/min for 10 min, and removed. The leaf area was scanned using a scanner (WinRHIZO La 2400, Epson, Japan), and the contents of K^+^, Na^+^, and Ca^2+^ in the leachate were determined using atomic absorption spectrophotometry (Agilent 240DUO, Thermo Fisher, USA). Each treatment was repeated thrice.

### Data analysis

The descriptive statistics and one-way analysis of variance (ANOVA) were conducted using SPSS 20.0 (IBM Corp., New York, USA) software. At a significance level of *P* < 0.05, Duncan’s multiple range tests were used to compare means. Data are presented as mean ± standard deviation, the OriginPro 2022b (Electron ic Arts Inc, New York, USA) software was used for plotting. The relationships between the measured indicators were analysed using principal component analysis (PCA) to obtain principal components based on eigenvalues > 1. All Indicators were examined using Pearson correlation analyses (OriginPro 2022b, Electron ic Arts Inc, New York, USA) for further interpretation.

## Results

### Effects of exogenous GB on the growth of salt-stressed *G. uralensis* seedlings

**Fig. 1:**
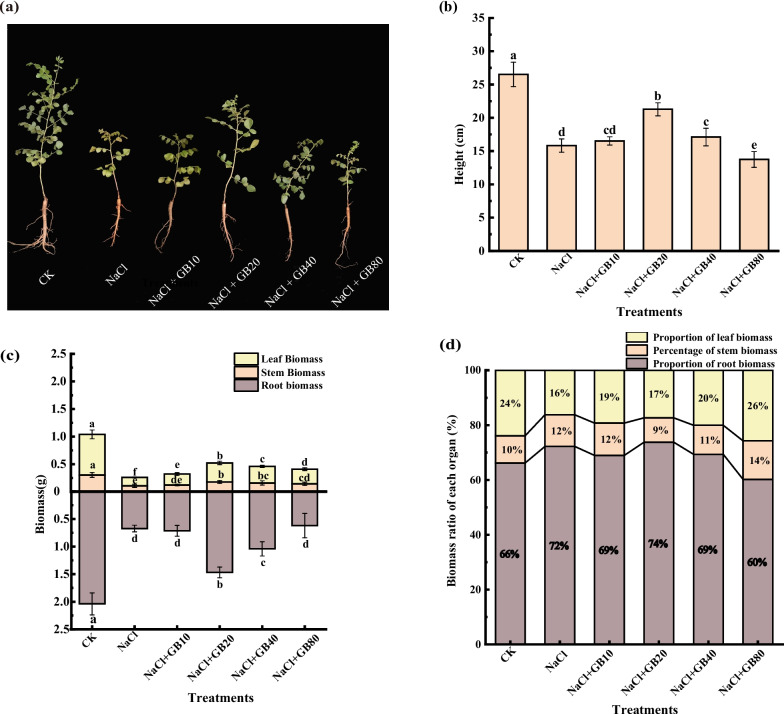
Effects of exogenous GB on (**a**) morphology (**b**) plant height, and (**c**) root, stem, and leaf biomass (**d**) root, stem, and leaf biomass percentage of *G. uralensis* seedlings under salt stress. Bars represent the SD of the mean; *n* = 10. Letters indicate significantly different values at *P* < 0.05 (Duncan’s method)

When the experiment was completed, phenotypic observations and images were collected for each treatment group. Plant height and root, stem, and leaf biomass were measured and counted. NaCl stress significantly inhibited the growth of *G. uralensis*, with significantly more yellow leaves and weaker individual growth on *G. uralensis* seedlings under NaCl stress than those in the control (Fig. 1a). Under the salt stress (NaCl) condition, the plant height (Fig. 1b) and the biomass of roots, stems, and leaves (Fig. 1c) of *G. uralensis* decreased by 40.31%, 66.99%, 64.76%, and 79.42%, respectively, compared with those CK, however the root biomass percentage increased from 66.21% in CK treatment to 72.23% in NaCl-treated group (Fig. 1d). GB significantly ameliorated the leaf yellowing and growth inhibition of *G. uralensis* seedlings caused by NaCl stress and promoted the accumulation of biomass in various organs. After the application of 20 mM GB, all the growth indices of *G. uralensis* reached their maximum value, and the plant height and the biomass of roots, stems, and leaves increased by 34.42%, 118.24%, 64.78%, and 128.22%, respectively, compared with those of the NaCl-only treatment (*P* < 0.05). In conclusion, exogenous application of GB significantly alleviated the inhibitory effect of NaCl stress on the growth and yield of roots, stems, and leaves of *G. uralensis*.

**Fig. 2:**
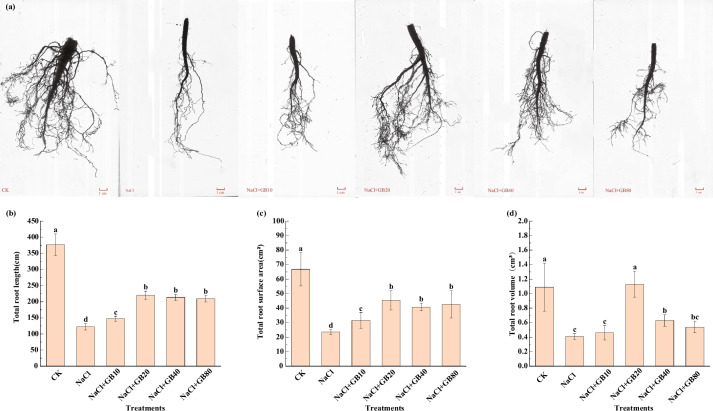
Effects of exogenous GB on (**a**) root morphology (**b**) total root length (TRL), and (**c**) total root surface area (TRSA), and (**d**) total root volume (TRV) of *G. uralensis* seedlings under salt stress. Bars represent the SD of the mean; *n* = 10. Letters indicate significantly different values at *P* < 0.05 (Duncan’s method)

Salt in the soil inhibited the root growth of *G. uralensis* by reducing its TRL (Fig. 2b), TRSA (Fig. 2c), and TRV (Fig. 2d), resulting in the root indices of NaCl-only treatment being significantly lower than those of CK (Fig. 2a) (*P* < 0.05). The application of 20 mM GB was able to effectively alleviate the inhibitory effect of salt damage on the root growth of *G. uralensis*. The TRL, TRSA, and TRV of *G. uralensis* increased by 80.38%, 93.28%, and 175.61%, respectively, compared to the NaCl-only treatment. This indicated that the GB treatment was able to promote the root growth of *G. uralensis* and thus alleviate the damage caused by the NaCl stress to the seedlings.

### Effects of exogenous GB on endogenous GB content of salt-stressed *G. uralensis* seedlings

**Fig. 3:**
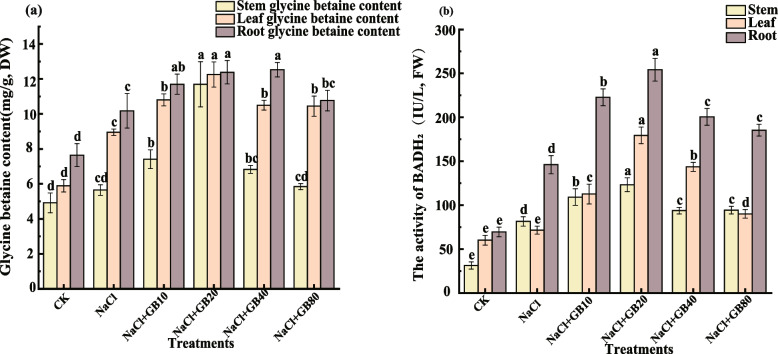
Effects of exogenous GB on (**a**) endogenous GB content and (**b**) BADH_2_ enzyme activity of *G. uralensis* seedlings under salt stress. Bars represent the SD of the mean; *n* = 3. Letters indicate significantly different values at *P* < 0.05 (Duncan’s method)

Endogenous GB content in roots, stems, and leaves of *G. uralensis* were obviously increased after salt stress treatment (Fig. 3a), and BADH_2_ activity was enhanced (Fig. 3b). The GB content and BADH_2_ enzyme activity were significantly increased in roots, stems, and leaves of *G. uralensis* after the application of exogenous GB. Especially, the GB content and BADH_2_ enzyme activity reached the maximum value after addition of 20 mM GB, in which the GB content in roots, stems, and leaves increased by 36.88%, 107.05% and 21.63%, respectively, compared with the NaCl-only treatment; the activity of the BADH_2_ enzyme increased by 74.10%, 249.38% and 150.60% compared with the NaCl-only treatment.

### Effects of exogenous GB on soluble protein, soluble sugar, and proline contents of salt-stressed *G. uralensis* seedlings

**Fig. 4:**
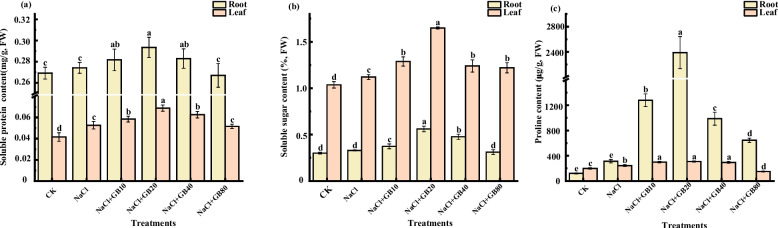
Effects of exogenous GB on (**a**) soluble protein, (**b**) soluble sugar, and (**c**) proline contents of *G. uralensis* seedlings under salt stress. Bars represent the SD of the mean; *n* = 3. Letters indicate significantly different values at *P* < 0.05 (Duncan’s method)

The contents of soluble protein (Fig. 4a), soluble sugar (Fig. 4b), and proline (Fig. 4c) in the roots and leaves of *G. uralensis* showed different degrees of increase after NaCl stress. Under salt stress conditions, the contents of soluble protein, soluble sugar, and proline in the roots and leaves of *G. uralensis* reached the maximum value after application of 20 mM GB; the contents of soluble protein, soluble sugar, and proline in roots increased by 7.05%, 70.52% and 661.06%, respectively, compared with those NaCl-only stress; and the three aforementioned osmotic regulators in the leaves increased significantly by 30.74%, 47.11%, and 26.88%, respectively. Thus, the GB treatment was able to significantly promote the synthesis of these osmoregulatory substances and thus alleviate the damage of NaCl stress on *G. uralensis* seedlings.

### Effects of exogenous GB on reactive oxygen metabolism systems of salt-stressed *G. uralensis* seedlings

**Fig. 5:**
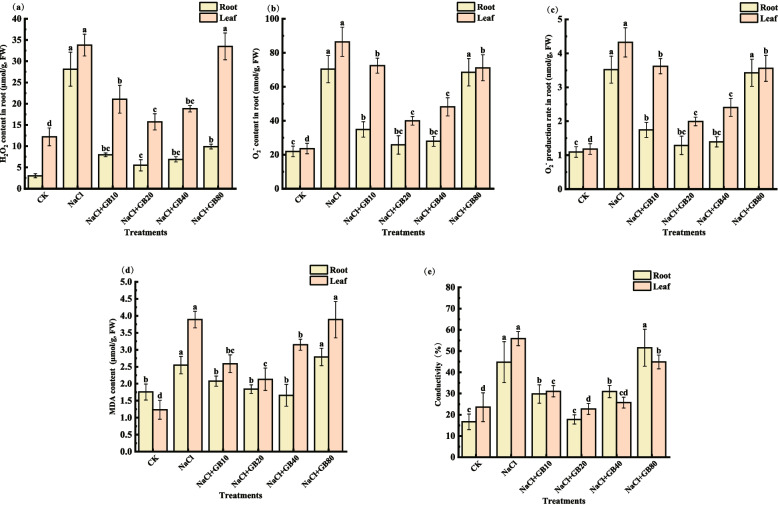
Effects of exogenous GB on (**a**) H_2_O_2_, (**b**) O_2_^−^^.^ content, (**c)** rate of O_2_^−^^.^ production, (**d**) MDA content, and (**e**) conductivity of *G. uralensis* seedlings under salt stress. Bars represent the SD of the mean; *n* = 3. Letters indicate significantly different values at *P* < 0.05 (Duncan’s method)

The H_2_O_2_ (Fig. 5a), O_2_^−^^·^content (Fig. 5b) and rate of O_2_^−^_._ production (Fig. 5c) of *G. uralensis* seedlings significantly increased under NaCl-only treatment compared with CK. Here, the three indices in roots increased by 825.07%, 221.26%, and 221.26%, and those in leaves increased by 213.5%, 177.12%, and 266.38%, respectively (*P* < 0.05). In contrast, the application of exogenous GB under salt stress decreased the H_2_O_2_ and O_2_^−·^ content and reduced the rate of O_2_^−^ production in roots and leaves. After the application of 20 mM GB, the H_2_O_2_, O_2_^−^_._ content and O_2_^−^^.^ production rate of the *G. uralensis* root system decreased by 80.49%, 63.41%, and 63.41%, respectively, compared with that of NaCl-only treatment, and the aforementioned three indices in the leaves decreased by 53.47%, 53.86%, and 53.86%, in that order. The results indicated that NaCl stress caused a large accumulation of ROS in *G. uralensis*, and the application of GB reduced the ROS content, with the most obvious effect of 20 mM GB treatment.

The MDA (Fig. 5d) content of the roots and leaves increased by 44.97% and 214.29% (*P* < 0.05), respectively, and the electrical conductivity (Fig. 5e) increased by 168.14% and 137.13% in the NaCl-only treatment as compared with CK. The application of 20 mM GB significantly reduced the MDA content and conductivity of roots and leaves by 27.62% and 45.20% (*P* < 0.05) for MDA content and 60.23% and 59.30% (*P* < 0.05) for conductivity content as compared with NaCl-only treatment. The results indicated that NaCl stress caused a significant increase in MDA content and relative conductivity in *G. uralensis*, and GB application was able to reduce MDA content and leaf relative conductivity, with the most pronounced reduction in the 20 mM GB treatment group.

**Fig. 6:**
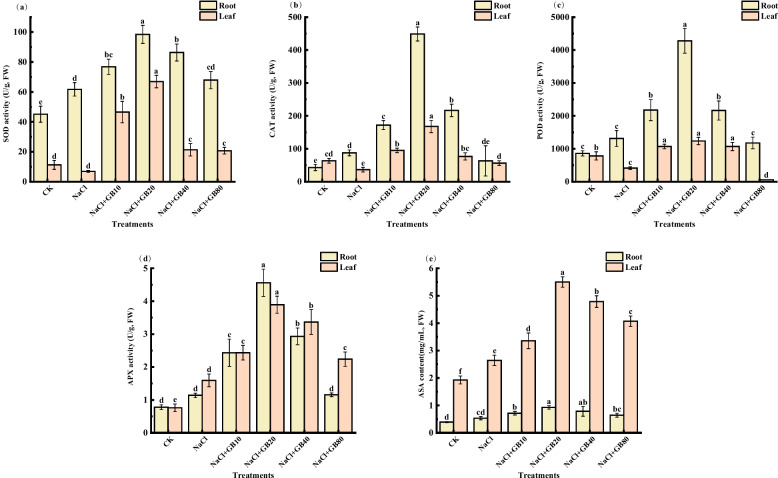
Effects of exogenous GB on (**a**) SOD, (**b**) CAT, (**c**) POD, (**d**) APX activities, and (**e**) ASA contents of *G. uralensis* seedlings under salt stress. Bars represent the SD of the mean; *n* = 3. Letters indicate significantly different values at *P* < 0.05 (Duncan’s method)

SOD (Fig. 6a), CAT (Fig. 6b), POD (Fig. 6c), APX (Fig. 6d) activities, and ASA (Fig. 6e) contents in the roots of *G. uralensis* were significantly higher than those of the control (increased by 36.86%, 103.55%, 52.60%, 46.15%, and 36.37%, respectively) under NaCl stress. The activities of SOD, CAT, and POD in leaves of *G. uralensis* were significantly lower than those of the control (decreased by 38.88%, 42.53%, and 47.33%, respectively); the APX activity and ASA content in the leaves were significantly higher than those of the control (increased by 109.65% and 37.04%, respectively). The maximum values of antioxidant enzyme activities and antioxidant contents were reached in roots and leaves of *G. uralensis* after the application of 20 mM GB, where SOD, CAT, POD and APX activities and ASA contents in roots were elevated by 59.55%, 413.07%, 225.91%, 300.00% and 73.33%, respectively, as compared with the NaCl-only treatment. The aforementioned five indices in leaves increased by 877.51%, 359.89%, 199.15%, 144.35%, and 108.11%, respectively.

### Effects of exogenous GB on salt secretion capacity of salt-stressed *G. uralensis* seedlings

**Fig. 7:**
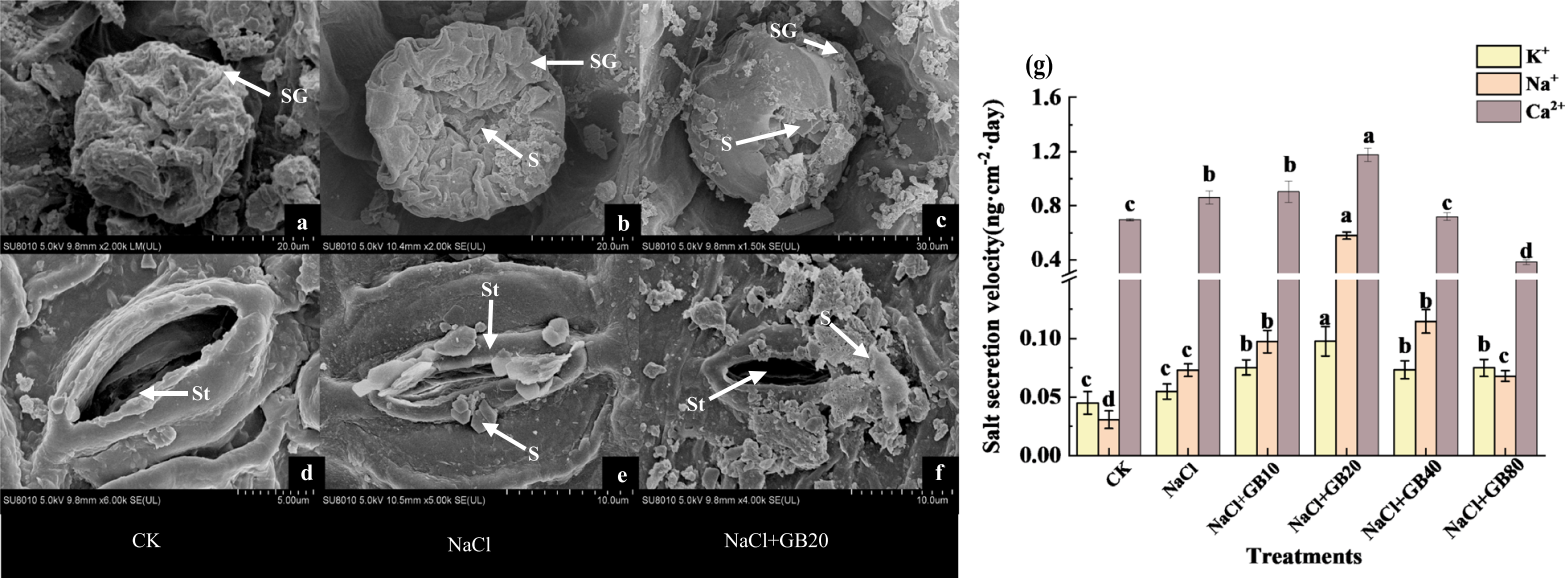
The effect of exogenous GB on (**a**) salt gland (SG) and stomata (St) morphology and (**b**) salt secretion from saline glands and stomata of *G. uralensis*. Bars represent the SD of the mean; *n* = 3. Letters indicate significantly different values at* P* < 0.05 (Duncan's method). Abbreviations stand for: Salt gland; St: Stoma; S: Salt

The salt glands and stomata on the leaves were observed, we did not find any salt crystals on the glands or around the stomata in the CK group (Fig. 7a, d). Under the NaCl stress conditions, salt secretion was observed in salt glands (Fig. 7b) and stomata (Fig. 7e). Under the NaCl stress condition, the salt secretion capacity of the stomata and salt glands was significantly enhanced (*P* < 0.05) after the application of 20 mM GB (Fig. 7c, f), and the secretion rates of Ca^2+^, Na^+^, and K^+^ were 1.79, 7.93, and 1.37 times higher than those of the NaCl-only treatment group, respectively (Fig. 7g).

### Trait correlations and variations

**Fig. 8:**
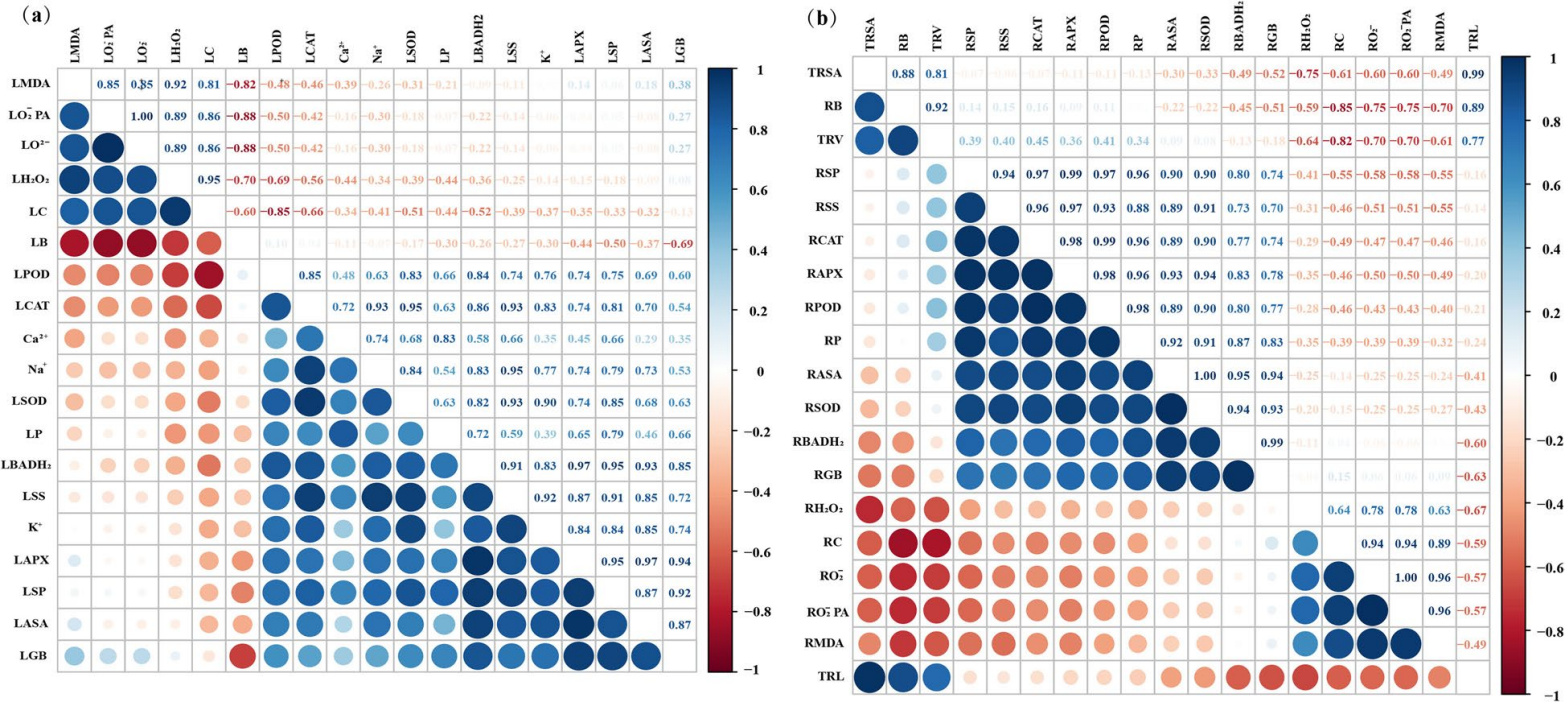
Correlation analysis between all the studied parameters among (**a**) aboveground and (**b**) above below traits. Blue and red color represent the positive and negative correlation. The size and intensity of color exhibited the significance of variables, and the significance level of correlations is indicated; abbreviations stand for: PH: plant height, RB: root biomass, SB: stem biomass, LB: leaf biomass, TRL: total root length, TRSA: total root surface area, TRV: total root volume, RGB: root GB content, SGB: stem GB content, LGB: leaf GB content, RBADH_2_: root betaine aldehyde dehydrogenase, SBADH_2_: stem betaine aldehyde dehydrogenase, LBADH_2_: leaf betaine aldehyde dehydrogenase, RSP: root soluble protein, LSP: leaf soluble protein, RSS: root soluble sugar, LSS: leaf soluble sugar, RP: root proline, LP: leaf proline, RH_2_O_2_: root hydrogen peroxide, LH_2_O_2_: leaf hydrogen peroxide, RO_2_^−^^.^: root superoxide anion, LO_2_^−^^.^: superoxide anion, RO_2_^−^^.^PA: root superoxide anion production rate, LO_2_^−^_._PA: leaf superoxide anion production rate, RMDA: root malondialdehyde, LMDA: leaf malondialdehyde, RC: root conductivity, LC: leaf conductivity, RSOD: root superoxide dismutase, LSOD: leaf superoxide dismutase, RCAT: root catalase, LCAT: leaf catalase, RPOD: root peroxidase, LPOD: leaf peroxidase, RAPX: root ascorbate peroxidase, LAPX: leaf ascorbate peroxidase, RASA: root ascorbic acid, LASA: leaf ascorbic acid, K^+^: potassium ion secretion, Na^+^: sodium ion, Ca^2+^: calcium ion

To effectively examine the response of *G. uralensis* leaves and roots to salt stress, we conducted Spearman's correlation analysis for each of the 19 representative traits of its leaves and roots, respectively. The results show the correlations among the indicators of *G. uralensis* leaves. Here, endogenous GB was significantly and positively correlated with BADH_2_, antioxidant enzymes (SOD, CAT, POD, and APX) activities, osmoregulatory substances (soluble sugars, soluble proteins, and proline), and salt secretion rate (Na^+^, K^+^, and Ca^2+^) (Fig. 8a).

Figure 8b shows the correlation among the indicators of *G. uralensis* roots, where endogenous GB in the root system was significantly and positively correlated with BADH_2_, antioxidant enzymes (SOD, CAT, POD, and APX) activities, and osmoregulatory substances (soluble sugars, soluble proteins, and proline).

**Fig. 9:**
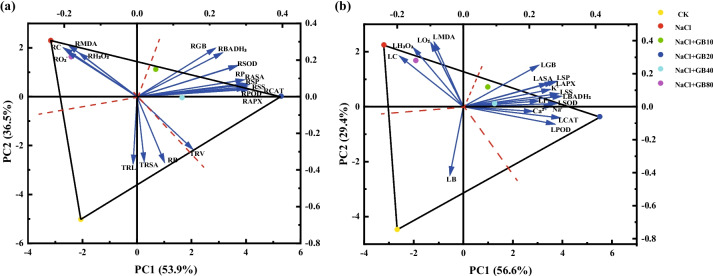
Principal component analysis (PCA) on the effects of exogenous GB on relative physiological indices in the root and leaf of *G. uralensi* seedling under salt stress. Abbreviations stand for: PH: plant height, RB: root biomass, SB: stem biomass, LB: leaf biomass, TRL: total root length, TRSA: total root surface area, TRV: total root volume, RGB: root GB content, SGB: stem GB content, LGB: leaf GB content, RBADH_2_: root betaine aldehyde dehydrogenase, SBADH_2_: stem betaine aldehyde dehydrogenase, LBADH_2_: leaf betaine aldehyde dehydrogenase, RSP: root soluble protein, LSP: leaf soluble protein, RSS: root soluble sugar, LSS: leaf soluble sugar, RP: root proline, LP: leaf proline, RH_2_O_2_: root hydrogen peroxide, LH_2_O_2_: leaf hydrogen peroxide, RO_2_^−^^.^: root superoxide anion, LO_2_^−^_._: superoxide anion, RO_2_^−^^.^PA: root superoxide anion production rate, LO_2_^−^_._PA: leaf superoxide anion production rate, RMDA: root malondialdehyde, LMDA: leaf malondialdehyde, RC: root conductivity, LC: leaf conductivity, RSOD: root superoxide dismutase, LSOD: leaf superoxide dismutase, RCAT: root catalase, LCAT: leaf catalase, RPOD: root peroxidase, LPOD: leaf peroxidase, RAPX: root ascorbate peroxidase, LAPX: leaf ascorbate peroxidase, RASA: root ascorbic acid, LASA: leaf ascorbic acid, K^+^: potassium ion secretion, Na^+^: sodium ion, Ca^2+^: calcium ion

Figure 9a shows the PCA of the effect of exogenous GB on physiological indices of *G. uralensis* seedling roots under NaCl stress, with the contribution value of the first and second principal components being PC1 = 53.9% and PC2 = 36.5%, respectively, and the two-dimensional plot reflecting 90.4% of the true information for each treatment. Triangles were formed by connecting the outermost treatment points (CK, NaCl, and NaCl + GB20). Three dashed lines perpendicular to the sides from the center point divided the whole graph into three regions. The root biomass (RB), TRL, and TRSA are shown in the region, which means that the RB, TRL, and TRSA of the CK group were significantly higher than those in other treatments. The region with NaCl treatment as the vertex also included NaCl + GB80 treatment, and the indices in this region were MDA, H_2_O_2_, O_2_^−·^, and O_2_^−·^ production rate, and all the angle between the index vectors were acute angles, indicating a positive correlation with each other. This result indicated that NaCl stress led to an increase in ROS content, enhanced membrane lipid peroxidation, and extravasation of intracellular substances in the root system of *G. uralensis*; therefore, the damage was greater than that in other treatments. By contrast, the indicators of antioxidant enzymes, antioxidants, and osmoregulatory substances were distributed in the region with NaCl + GB20 treatment as the apex, and the region also included the NaCl + GB10 treatment and NaCl + GB40 treatment. This phenomenon showed that the addition of 20 mM GB treatment under NaCl stress had the highest antioxidant enzyme activity, antioxidant content, and osmoregulatory substances, which reduced the degree of peroxidation of membrane lipids and mitigated the negative effects of NaCl stress on the root system of *G. uralensis*, and that the positive correlation and the coordinated effect among the indices were able to better scavenge the excessive ROS produced by NaCl stress and stabilize the balance of cellular oxidative metabolism.

The PCA of leaf-related physiological indices is shown in Fig. 9b. The information loading of the first and second principal components were PC1 = 56.6% and PC2 = 29.4%, respectively, and the two-dimensional graph reflected 86.0% of the true information for each treatment. Connecting the vertices of each treatment in the outermost (CK, NaCl, and NaCl + GB20), vertical dashed lines were used for each side to divide the whole graph into three regions. Among them, MDA, H_2_O_2_, O_2_^−^_._, and O_2_^−^^.^ production rates were distributed in the region with NaCl treatment as the vertex. There was an acute angle between the index vectors, which suggested a positive correlation. Therefore, the MDA, H_2_O_2_, and O_2_^−·^content of *G. uralensis* leaves was relatively high under NaCl treatment, and the degree of damage was greater than that of the other treatments. Indicators distributed within the region of CK were leaf biomass, which indicated that *G. uralensis* leaf biomass was the highest in the CK treatment. The NaCl + GB10 treatment and NaCl + GB40 treatment were also included in the region with NaCl + GB20 treatment as the apex. The indicators of antioxidant enzymes, antioxidants, osmoregulatory substances, and salt secretion rate were distributed in this region; moreover, the antioxidant enzyme activities were improved, osmoregulatory substances were increased. The secretion rate of leaves was promoted under the 20 mM GB treatment. Under NaCl stress, the ROS in the leaves of *G. uralensis* increased. This led to the increase in membrane lipid peroxidation and disrupted the oxidative metabolism equilibrium; meanwhile, the addition of GB was able to increase the activities of antioxidant enzymes and the contents of antioxidants and osmotic regulators and promoted the secretion of salts by the leaves. This alleviated the stress damage and stabilised the oxidative metabolism level.

## Discussion

Salt stress often inhibits plant growth through ionic toxicity, oxidative stress, and osmotic stress [37–40], and the strength of its inhibitory effects is directly reflected by growth indicators. In the present study, salt stress led to impaired growth features (plant height, biomass, root length, root surface area, and total root volume; Figs. 1 and 2). Additionally, *G. uralensis* exhibited a series of morphophysiological adjustments, including allocating more biomass to the root system, adapting to the salinity environment. In the NaCl stress group, the RB percentage increased from 66.21% in CK to 72.22%. In addition to increasing the percentage of below-ground biomass to adapt to the adversity, exogenous application of different concentrations of GB alleviated the growth inhibition of *G. uralensis* seedlings by salt stress. The inhibition of root growth is the most obvious characteristic of plants under salt stress at its early stage [41, 42]. Root length, root surface area, and root volume are indicators of root growth and development [43]. Shen (2022) found that NaCl stress inhibited the growth of the root system of *G. uralensis* and that the application of silicon was able to attenuate the detrimental effects of salt stress [44]; these results are similar to ours. We found that GB promoted the elongation of primary roots and the growth of lateral roots of *G. uralensis* under NaCl stress. Leading to a significant increase in the total length, total surface area, and volume of the root system. Thus, the exogenous GB expanded the range of water uptake and increased the efficiency of water and mineral absorption by the roots of *G. uralensis* under NaCl stress, which mitigated the adverse effects of salt stress. In contrast with other cash crops such as cotton (*Gossypium hirsutum* L.) [45] and maize (*Zea mays* L.) [46], widely grown in arid regions, the root system is the main economic organ of *G. uralensis*. Its growth status has a direct impact on the economic income of the farmers. The growth condition of the root system, especially the length and thickness of the main root, is a key indicator of the quality grade of liquorice herbs, and the thicker and longer the main root, the higher the grade of the herb [47]. The results of this study showed that exogenous GB could significantly promote the elongation and thickening of the primary roots of *G. uralensis* seedlings under NaCl stress. This is conducive to the enhancement of the herb grade and plays a role in increasing the herb production and farmer’s income.

Exogenous application of GB increased the synthesis of endogenous GB. BADH_2_ is the rate-limiting enzyme in the GB biosynthesis pathway [48]. Our results showed that with the increase of the concentration of exogenous GB, the content of endogenous GB in the roots, stems and leaves of *G. uralensis* increased first and then decreased, and higher than the NaCl-only treatment (Fig. 3a). We speculated that the increase of GB content in *G. uralensis* includes not only the absorption of exogenous GB but also the synthesis of endogenous GB. To verify the effect of exogenous GB on endogenous GB synthesis, the enzymatic activity of BADH_2_ was measured in our experiment. The data showed exogenous GB at different concentrations could significantly increase the activities of BADH_2_ (Fig. 3b). However, it is not necessarily the case that a higher concentration of exogenous GB yields superior results. The activity of the BADH_2_ and the content of endogenous GB e increased first and then decreased. This indicates that the inhibition of endogenous GB synthesis due to the high concentration of exogenous GB [49]. The accumulation of endogenous plant betaine adversity stress is crucial for cellular osmoregulation [50]. Currently, there are three main pathways for the accumulation of endogenous betaine in plants: Firstly, the synthesis and accumulation of betaine are induced in plants by drought [51], salt [52], and heavy metal stress [50]; Secondly, genes for key enzymes of betaine synthesis (e.g., BADH genes) are introduced into plants by using transgenic technology to equip plants with the ability to synthesize more endogenous betaine [53]; Thirdly, the synthesis and accumulation of endogenous betaine in plants is induced by chemical inducers such as inducing endogenous betaine synthesis and accumulation in plants through application of exogenous betaine [54]. Several similar studies have demonstrated that exogenous GB can promote the accumulation of endogenous GB in different crops under stress conditions. For example, Ji (2022) found that Pb stress induced the accumulation of endogenous GB in roots and shoots of pakchoi (*Brassica chinensis* L.), and the induced effect was more pronounced when 5 mM GB was applied under Pb stress [50]. In our study, we observed similar findings: NaCl stress significantly induced endogenous betaine accumulation in roots, stems, and leaves of *G. uralensis*, and root application of exogenous GB significantly increased the endogenous betaine content of *G. uralensis*.

BADH_2_ is the rate-limiting enzyme for endogenous GB synthesis in plants [55]. In this study, exogenous application of GB was able to effectively increase the activity of BADH_2_, and it could be responsible for promoting endogenous glycine betaine accumulation [56]. Hence, we suggest that exogenous GB could increase the salinity-tolerance of *G. uralensis* by improving their endogenous betaine content and the activity of BADH_2_ under salt stress.

Under salt stress, the osmotic potential around the root system decreases, and osmoregulatory substances (e.g., soluble sugars, soluble proteins, free proline) are rapidly produced in the plant to avoid a large amount of water loss from the root cells [57]. This helps ensure a normal water supply to the plant to manage with the salt-stressed environment. Proline and soluble sugars are two important osmoregulatory substances that enable water transport across membranes in a direction that favours plant growth [58, 59]. Most of the soluble proteins in plants are enzymes involved in metabolism, and their content reflects the intensity of plant metabolism. Additionally, soluble proteins have strong hydrophilicity, which can improve cellular water retention performance and effectively prevent dehydration of the plant root system [60]. The results of this study showed that the content of soluble sugars, soluble proteins, and free proline in the roots and leaves of *G. uralensis* under NaCl stress were significantly higher than those of the CK. The large-scale synthesis of osmotic adjustment substances may be to help in the regulation of osmotic balance and stabilizing biological macromolecule structure under stress [61]. Li et al. also reported that GB increased the soluble protein content in maize exposed to stress by effectively reducing protein carbonylation and promoting protein synthesis [62]. We found that application of GB to NaCl-stressed *G. uralensis* plants considerably increased the levels of proline, soluble sugar, and protein, so as to reduce the salinity damage to *G. uralensis* growth, resulting increased biomass (Fig. 4).

Under normal physiological conditions, the generation and removal of ROS in plants are a dynamic equilibrium, and ROS is maintained at a low level [63]. When subjected to severe salt stress, the structure and function of chloroplasts are damaged, and the reduction of CO_2_ assimilation capacity causes a large amount of electron transfer to O_2_^−^_._, resulting in the excessive accumulation of ROS in the plant. Those ROS will attack the electron transport chain, destroy the structure of proteins and other biomolecules, and cause oxidative damage [64].The O_2_^−^^.^, H_2_O_2_, and MDA contents were significantly increased in roots and leaves of *G. uralensis* seedlings under NaCl stress in this study (*P* < 0.05) (Fig. 5), which indicated that salinity may cause oxidative damage to *uralensis*. Similar result was reported by Aazami et al. where salinity induced excess ROS in tomato [37]. Application of GB remarkably declined the O_2_^−^, H_2_O_2_, and MDA contents both in in roots and leaves of *G. uralensis* seedlings under NaCl stress (*P* < 0.05) (Fig. 5). Exogenous GB may be as a direct antioxidant to reduce the content of ROS. Therefore, the reduction of ROS might be due to the enhanced accumulation of GB, which maintains the stability of cell membrane in *G. uralensis* [50].

Plants have evolved specific scavenging systems to avoid the negative effects of ROS, and improving the antioxidant metabolic capacity to scavenge ROS is crucial for plant resistance to salt stress [65]. The key factors in these processes are the enzymes that catalyst the metabolic reactions, among which SOD, POD, CAT, and APX are key antioxidant enzymes relevant to plant resistance [66]. For SOD, an important component, its main function is to disproportionate negative oxygen ions, produce H_2_O_2_ and O_2_^−^_._, and reduce the damage of ROS to the cells of a plant [56]. POD and CAT are common oxidoreductase enzymes in plant and important endogenous ROS scavengers in a cell. This enables the plant cells to alleviate the oxidative damage of ROS to a certain extent [37]. APX is an important enzyme involved in the ASA-GSH cycle, which catalyst the reaction between ASA and H_2_O_2_, scavenging excess H_2_O_2_ in plants [67]. In addition, ASA is an important non-enzymatic antioxidant, and a reactive oxygen scavenger produced during intracellular glucose metabolism. The degradation of H_2_O_2_ in plants is mainly accomplished by ASA, which also acts as a cofactor for some antioxidant enzymes, and feedback regulates antioxidant enzyme activities [67]. Therefore, maintaining a relatively stable level of ASA in plants under salt stress to maintain the normal physiological metabolism of cells and resist salt stress damage is critical. Plants maintain the balance of ROS metabolism mainly by regulating the activities of the key aforementioned antioxidant enzymes antioxidants. This reduces the salt stress triggered is intracellular reactive oxygen radical accumulation and membrane lipid peroxidation injury triggered by salt stress. Improving the antioxidant capacity of plants is a key prerequisite for plant growth in stressful environments [50]. Betaine plays an important role in antioxidant defending and ROS scavenging to protect plants from damage induced by oxidative stress, the finding was confirmed in studies on crops such as cotton (*Gossypium hirsutum* L.) [68] and lettuce (*Lactuca sativa*) [69], and the similar results were found in our results. Several studies have reported that the increase in enzyme activities may result from the regulation of genes encoding antioxidant enzymes and alleviate oxidative stress after GB treatments [70, 71], which corroborate our finding. In addition, GB does not function as an antioxidant and cannot directly curb ROS accumulation, but rather indirectly by activating the antioxidant enzyme system [72]. For example, Wang et al. (2016) found that exogenous GB produced more antioxidant enzymes in response to cold stress by promoting the transcription of POD, CAT, APX, GR (glutathione reductase), and LOX (lipoxygenase) in a cold-damaged treatment of chilli pepper (*Capsicum annuum* L. cv. Mutianqiushuo) [73]. Cisse, by contrast, demonstrated that exogenous GB increased the activities of SOD, POD, CAT, and APX in *Dalbergia odorifera* leaves under salt stress conditions. Nomura (1998) suggested that GB acts as a chaperone molecule that binds to enzyme proteins and enhances enzyme protein conformation in response to adversity stress in plants, further demonstrating that exogenous GB has a positive physiological regulatory effect on enhancing salt tolerance in plants [74].

Halophytes often have evolved a series of unique salt tolerance mechanisms to adapt to saline soil habitats. Majority of salt-secreting plants have salt-secreting structures, such as salt glands or salt vesicles, distributed on the stems and leaves, which are conducive to the organism's ability to excrete excess salts from the body [75, 76]. For example, *Limonium bicolor* L., a salt-secreting saline plant of the Plumbaginaceae, showed a positive correlation between the rate of salt gland secretion and soil salt concentration. The rate of Na^+^ secretion by salt glands in the leaves of *Limonium bicolor* seedlings reached a maximum under 400 mM NaCl stress [77]. In addition to its salt glands, the stomata of *G. uralensis* leaves also function as salt secretion [35]. These were also observed by scanning electron microscopy in this study. Using atomic absorption split-flame spectrophotometry to determine the content of ions on the surface of *G. uralensis* leaves, we found that the rate of leaf salt-secreting was significantly higher than that of the control group under NaCl stress, which implies that *G. uralensis* can excrete excessive salts from its body to alleviate the salinity-damaging effect under salt stress. The regulation the rate of salt secretion by exogenous substances in saline plants has become a key research focus. For example, Li (2020) used a Nikon fluorescence microscope and flame spectrophotometer to observe the morphology of salt glands of *Limonium bicolor* L. and measure the rate of Na^+^ excretion and found that the addition of exogenous melatonin (MT) significantly increased the number of salt glands on its leaves and the radius of salt glands and promoted the rate of Na^+^ excretion from salt glands, which effectively improved its salt tolerance [78]. In addition, Wei (2022) found that exogenous hydrogen sulfide (H_2_S) treatment increased the rate of Na^+^ excretion from the salt glands of the mangrove plant *Avicennia marina* (Forsk) under salt stress conditions and found that H_2_S promoted Na^+^ secretion from the salt glands through the upregulation of Na^+^/H^+^ antagonists and H^+^-ATPase in the salt glands' plasma membranes and chromatophores as detected by qRT-PCR [79]. In our experiment, we observed the same phenomenon as aforementioned, with a significant increase in salt secretion around the salt glands and stomata of GB-treated *G. uralensis*. This may be attributed to the regulatory effect of exogenous GB on stomatal opening and closing [67]. When leaf leachate was examined using the atomic absorption flame spectrophotometer method, we found that the content of Na^+^, K^+^, and Ca^2+^ in the GB-treated group were significantly higher than that in the salt stress treatment, especially after the application of 20 mM GB. The rate of leaf secretion of Na^+^ was 7.93 times higher than that of the NaCl-only treatment, which implies that the exogenous GB reduces the Na^+^ accumulation in the plant by accelerating the rate of leaf secretion. However, it is unclear which pathway of betaine promotes the secretion of salts in plant leaves. We hypothesis that the ability of exogenous GB to promote salt secretion in liquorice may be related to the involvement of GB in regulating the morphology of leaf stomata [52] and salt glands.

## Conclusion

**Fig. 10:**
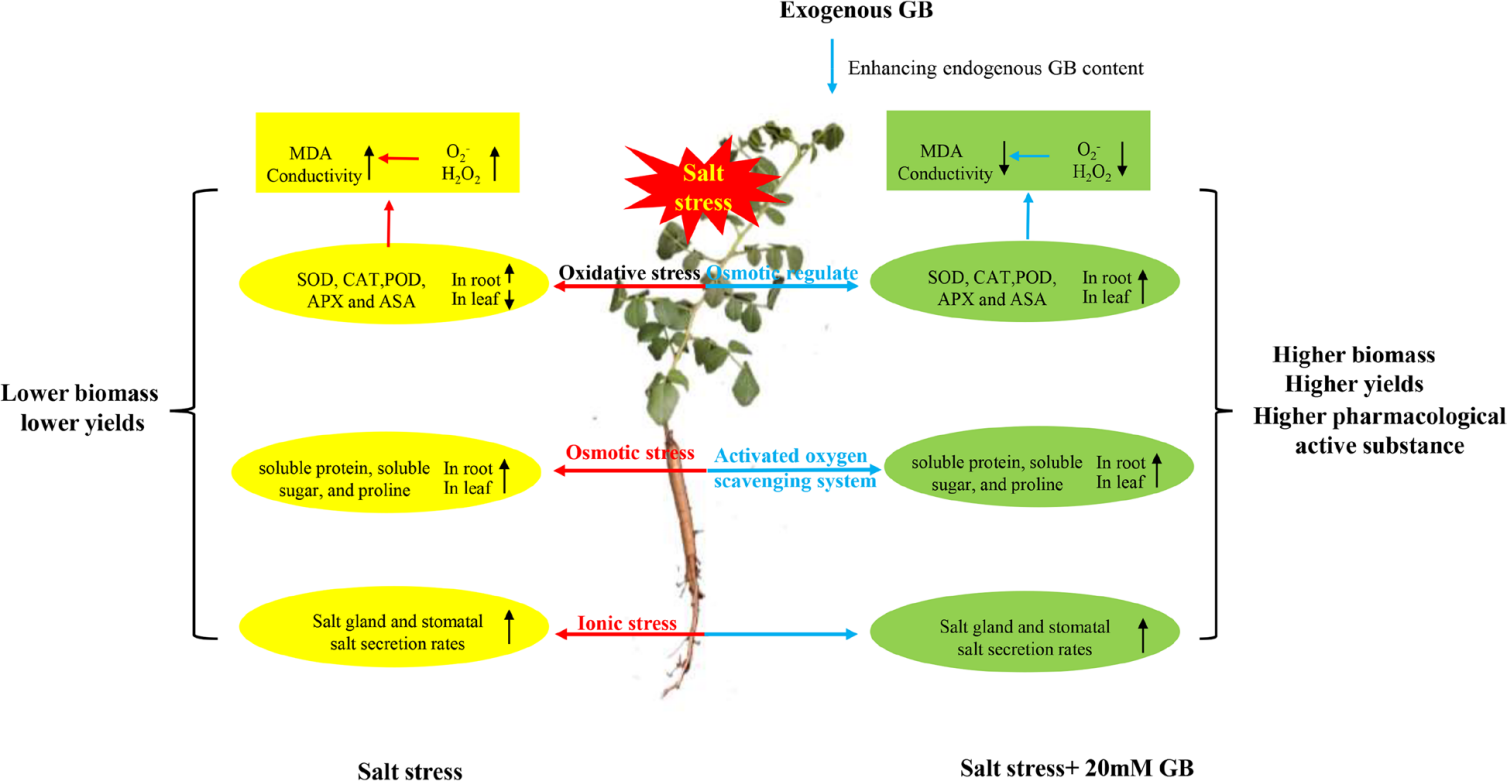
Summary of exogenous betaine on salt damage mitigation in *G. uralensis* under salt stress conditions

In summary, to determine how exogenous GB mitigates the adverse effects of salt stress on *G. uralensis* seedlings growths, we summarised the potential mechanisms of its action. In this study, NaCl stress reduced the biomass of *G. uralensis*. Exogenous application of GB can further increased the biomass accumulation of *G. uralensis* and enhanced the salt tolerance of *G. uralensis* seedlings by increasing the synthesis of endogenous betaine, antioxidant enzyme activity, osmoregulatory capacity, and leaf salt secretion capacity (Fig. 10). In conclusion, the results of this experiment proved that exogenous betaine had the positive effect on the growth of *G. uralensis* seedlings and the enhancement of herb yield and quality, and the best characterization effect was at 20 Mm GB. This study first indicated the important role of GB in influencing *G. uralensis* growth, offering a theoretical basis for exogenous GB to alleviate NaCl stress in *G. uralensis* and a scientific basis for the development of high-yield and high-quality cultivation technology of liquorice herbs by using salinized soil. Future research should investigate whether exogenous GB can positively influence the saline stress tolerance of *G. uralensis* at the molecular mechanisms to offer solutions to its low productivity in salt-affected areas.

## Data Availability

No datasets were generated or analysed during the current study.

## Abbreviations

PH: Plant height
RB: Root biomass
SB: Stem biomass
LB: Leaf biomass
TRL: Total root length
TRSA: Total root surface area
TRV: Total root volume
RGB: Root GB content
SGB: Stem GB content
LGB: Leaf GB content
RBADH_2_: Root betaine aldehyde dehydrogenase
SBADH_2_: Stem betaine aldehyde dehydrogenase
LBADH_2_: Leaf betaine aldehyde dehydrogenase
RSP: Root soluble protein
LSP: Leaf soluble protein
RSS: Root soluble sugar
LSS: Leaf soluble sugar
RP: Root proline
LP: Leaf proline
RH_2_O_2_: Root hydrogen peroxide
LH_2_O_2_: Leaf hydrogen peroxide
RO_2_^−^^.^: Root superoxide anion
LO_2_^−^^.^: Superoxide anion
RO_2_^−^^.^ PA: Root superoxide anion production rate
LO_2_^−^^.^ PA: Leaf superoxide anion production rate
RMDA: Root malondialdehyde
LMDA: Leaf malondialdehyde
RC: Root conductivity
LC: Leaf conductivity
RSOD: Root superoxide dismutase
LSOD: Leaf superoxide dismutase
RCAT: Root catalase
LCAT: Leaf catalase
RPOD: Root peroxidase
LPOD: Leaf peroxidase
RAPX: Root ascorbate peroxidase
LAPX: Leaf ascorbate peroxidase
RASA: Root ascorbic acid
LASA: Leaf ascorbic acid
K^+^: Potassium ion secretion
Na^+^: Sodium ion
Ca^2+^: Calcium ion
